# Modelling and simulation of complex sociotechnical systems: envisioning and analysing work environments

**DOI:** 10.1080/00140139.2015.1008586

**Published:** 2015-03-11

**Authors:** Lawrence J. Hettinger, Alex Kirlik, Yang Miang Goh, Peter Buckle

**Affiliations:** ^a^ Liberty Mutual Research Institute for Safety, 71 Frankland Road, Hopkinton, MA01748, USA; ^b^ Thomas M. Siebel Center for Computer Science, University of Illinois, 201 N. Goodwin Avenue, Urbana, IL61801, USA; ^c^ School of Design and Environment, National University of Singapore, Singapore, Singapore; ^d^ Royal College of Art, Kensington Gore, London, UK

**Keywords:** sociotechnical systems, modelling and simulation, occupational safety, complex adaptive systems

## Abstract

Accurate comprehension and analysis of complex sociotechnical systems is a daunting task. Empirically examining, or simply envisioning the structure and behaviour of such systems challenges traditional analytic and experimental approaches as well as our everyday cognitive capabilities. Computer-based models and simulations afford potentially useful means of accomplishing sociotechnical system design and analysis objectives. From a design perspective, they can provide a basis for a common mental model among stakeholders, thereby facilitating accurate comprehension of factors impacting system performance and potential effects of system modifications. From a research perspective, models and simulations afford the means to study aspects of sociotechnical system design and operation, including the potential impact of modifications to structural and dynamic system properties, in ways not feasible with traditional experimental approaches. This paper describes issues involved in the design and use of such models and simulations and describes a proposed path forward to their development and implementation.

**Practitioner Summary:** The size and complexity of real-world sociotechnical systems can present significant barriers to their design, comprehension and empirical analysis. This article describes the potential advantages of computer-based models and simulations for understanding factors that impact sociotechnical system design and operation, particularly with respect to process and occupational safety.

## 1. Introduction

Essentially, all models are wrong, but some are useful. (Box and Draper 1987, 424)

The concept of the sociotechnical system originated with the insights of Tavistock Institute researchers in the early 1950s, specifically with respect to examining the impact of the introduction of novel technical systems in the British coal mining industry (e.g. Trist and Bamforth 1951). Although it remained a largely under-appreciated domain throughout the 1960s and 1970s, the sociotechnical ‘movement’ re-emerged in the 1980s and beyond with the advent of several important theoretical and practice-oriented approaches. These include areas such as cognitive systems engineering (Hollnagel and Woods 1983; Rasmussen, Pejtersen, and Goodstein 1994), macroergonomics (Hendrick 1984; Hendrick and Kleiner 2002), Leveson's system theoretic accident model and processes approach (Leveson 2012), human-systems integration^4^ (Booher 2003; Tainsh 2004; Pew and Mavor 2007) and resilience engineering (Hollnagel, Woods, and Leveson 2006).

As discussed by Carayon et al. (2015), these approaches share a common theoretical framework that focuses on the interactive influences of social-organisational and technical factors (hence, the hybrid term ‘sociotechnical’) as they impact the design and performance of complex operational systems. Social-organisational factors include system attributes related to personnel characteristics and organisational structure, policies and procedures. These can include features such as the number and types of people employed by an organisation and/or assigned to a particular job function, the structure of the organisational elements (i.e. the ‘organisational chart’) and the patterns of formal and informal command and control relationships that exist within it. Social factors also include features such as the nature of rewards (e.g. rate of pay, bonuses, criteria for promotion, etc.) and punishments (e.g. demotions, terminations, etc.) that play a central role in a worker's sense of what constitutes desirable and undesirable behaviour and accomplishments on the job. Technical factors include both the technical systems and tools that support the execution of work-related activity, as well as the technical processes and techniques used in its execution (see also Mumford 2006).

While the focus of this article and special issue is on workplace safety, sociotechnical factors are at the foundation of nearly all forms of contemporary human activity. Education, healthcare, entertainment and social media are among the many current, and frequently intersecting, examples of complex sociotechnical systems. Among the many domains currently exploring the use of a sociotechnical framework to help drive improvements in system performance and safety are medical systems design (e.g. Carayon et al. 2006; Challenger, Clegg, and Shepherd 2013; Holden et al. 2013), oil and natural gas production (e.g. Widdowson and Carr 2002), maritime search and rescue (e.g. Baber et al. 2013) and military systems design (e.g. Booher 2003; Stanton 2014).

There are several critical insights shared by all sociotechnical theories, each of which illuminates the common themes of a sociotechnical systems approach (Carayon et al. 2015). A number of these are particularly critical for envisioning the ways in which modelling and simulation can potentially support the design and analysis of real-world systems, as well as the scientific examination of general principles of sociotechnical systems. These include the broad acknowledgement of the complex nature of social and technical factors impacting system performance; the interactive, hierarchical nature of sociotechnical systems and the assertion that traditional experimental paradigms may not be equipped to adequately examine or explain complex system performance

Within sociotechnical systems theory, system-level performance outcomes are considered to arise as *emergent properties* of the interactions within and between system components. Safety, efficiency, profitability and other similar outcomes and attributes are considered to emerge as a function of the complex pattern of interactions within and between a system's social-organisational and technical components. This is a view shared by those social scientists who approach the study of economic, political, anthropologic and other social systems from the related perspective of complexity theory (e.g. Miller and Page 2007) and is a perspective familiar to biologists, physicists and others (Holland 2012).

In highly complex sociotechnical work systems, the majority of component interactions may not be readily apparent upon even quite detailed examination of system design and usage parameters, an issue that can present opportunities for unforeseen interactions that may, under the right circumstances, contribute to undesired and potentially risky outcomes (Leveson 2012). As stated by Hollnagel (2009): ‘The idea of a sociotechnical system is that the conditions for successful organizational performance – and conversely also for unsuccessful performance – are created by the interaction between social and technical factors’ (19). In the disciplines supporting sociotechnical system design and deployment, modelling and simulation afford a useful means for reducing risks associated with unforeseen, negative interactions and the ‘law of unintended consequences’ (e.g. Tenner 1997). In the scientific study of such systems, modelling and simulation are potentially powerful methods for uncovering the general principles that underlie such phenomena.

Work-based systems, and complex, adaptive systems in general (e.g. Holland 2012), are characterised by hierarchical structures consisting of numerous interacting sub-systems (see also Flach et al. 2015). The performance of ‘local’ work systems or processes, such as an individual industrial plant, construction site, etc., is considered by sociotechnical theorists to be continually impacted by the activities of larger and more diffuse systems through complex, nested patterns of communication and control. In turn, activities within and outcomes from local work systems can have reciprocal influences on these superordinate structures as, for example, when a significant workplace accident or disaster results in higher-level legislative, regulatory and/or policy changes.

**Figure 1:**
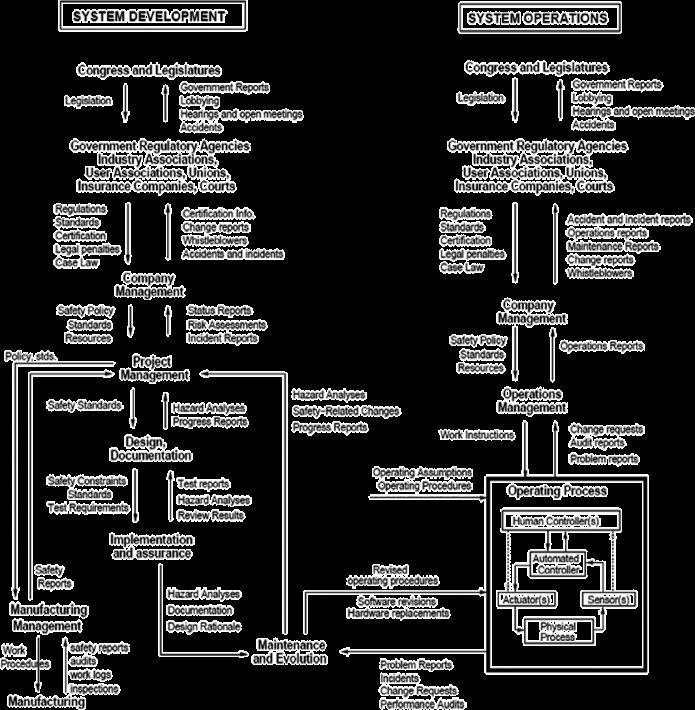
Hierarchical relations of organisational and technical systems impacted and impacted by local system activity (Leveson 2012).

Figure 1, taken from Leveson (2012) provides a conceptual illustration of this hierarchical set of relationships within sociotechnical systems. The figure depicts the various levels of legislative, regulatory, managerial and local work entities and sub-systems that impact and are in turn impacted by one another's activities. One can also conceive of similar, albeit more indirect, reciprocal influences between work systems and entities outside the boundary of the system as depicted in Figure 1. These include stakeholder groups such as customers, suppliers, ‘public opinion’ as manifest in the form of community and special interest groups, the media, etc. These are entities that are, in most respects, relatively autonomous from the sociotechnical system of interest, but whose activities are, nevertheless, impacted by those of the system under consideration and which, in turn, can exert reciprocal influences.^5^

Sociotechnical systems are considered to be formally *complex* in that their emergent properties and associated phenomena can have multiple causes and consequences (many of which are unforeseen and unintended) that are highly context dependent, difficult to predict and which frequently emerge unexpectedly from the many dynamic, non-linear relationships among system components. Therefore, sociotechnical systems are considered to be included among the general class of phenomena referred to as complex, adaptive systems (e.g. Lansing 2003; Miller and Page 2007).

Also common to sociotechnical perspectives is the view that traditional, reductionist approaches to the investigation of work systems, or any complex system in which human behaviour plays an important role, will generally only account for a small amount of the variance in factors impacting safe and effective performance (Waterson et al. 2015). Indeed, as discussed above, sociotechnical systems theory adopts a causal framework that is, in many ways, the opposite of that which has formed the basis of traditional psychological and social scientific research for many decades. Specifically, the traditional, epistemological assumption that empirical knowledge is best obtained by strict, experimental control of potentially confounding variables in order to assess the effect of one or several independent variables runs counter to the very nature of most thinking about complex systems (e.g. Wagner 1999). As Leveson (2012) notes, one of the more problematic outcomes of this traditional assumption has been the historical tendency of researchers and accident investigators to search for and identify ‘root causes’ of workplace accidents and disasters, i.e. one or more apparently discrete casual factors to which the occurrence of an accident can be attributed. While this bias can often result in the commonly-flawed attribution of ‘human error’ as a root cause for so many accidents and disasters (e.g. Reason 1998; Dekker 2006), it also tends to inhibit examination of the less immediately apparent and occasionally more diffuse, but critical influences of systemic, sociotechnical factors. A further negative outcome, more focused on research as opposed to accident investigation, has been the historical tendency of human factors and ergonomics researchers to limit the scope of their work to phenomena that can be carefully controlled in the traditional experimental sense. Of necessity, this generally precludes the ability to examine phenomena within the context of the larger sociotechnical settings within which salient sociotechnical factors exert their influence.

While not an exclusively sociotechnical theme, the concept of participatory or user-centred design is employed by nearly all sociotechnical theorists to refer to the notion that system design and deployment is best served through the consistent inclusion of input from system users and stakeholders (e.g. Noro and Imada 1991; Flach and Dominguez 1995). Interestingly, the importance of participatory design may extend to the very processes of modelling and simulation that seek to clarify issues involved in the design and operation of sociotechnical systems. For example, Sterman (2000), speaking from the perspective of system dynamics modelling, notes that models developed without the direct participation of stakeholders, such as managers, workers, etc., are unlikely to be either valid or useful.

While there are clear variations in emphasis and application among existing sociotechnical schools of thought, the concepts described above are commonly held and central to any sociotechnical approach to the study of complex human-machine and organisational systems. While we believe there is significant benefit in employing a sociotechnical approach to examine factors that underlie many different aspects of system performance (e.g. efficiency, profitability, etc.), we have chosen, within the spirit of this special issue, to focus on issues related to occupational safety. That being said, it is a fundamental tenet of sociotechnical approaches that performance and safety are each essential aspects of complex systems, and factors that influence one will, in nearly all cases, have significant implications for the other (e.g. Dul et al. 2012).

### 1.1. Sociotechnical systems, emergence and safety

We have suggested that sociotechnical systems are examples of the broad class of phenomena referred to as complex, adaptive systems. One of the more scientifically interesting and, from a practical perspective, important aspects of such systems is the concept of emergence, briefly discussed above. Goldstein (1999) has defined emergence as ‘the arising of novel and coherent structures, patterns and properties during the process of self-organization in complex systems’ (49). The concept of emergence upon which this definition rests is fundamental to the broader theoretical framework of general systems theory (von Bertalanffy 1968) and more recent theoretical treatments of complex, adaptive systems (e.g. Miller and Page 2007; Holland 2012) in general. From the perspective of our concern with occupational safety, an ‘emergence perspective’ would assert that safety cannot accurately be said to be ‘a product of’ or to ‘reside within’ one or more of the social and/or technical components of a work system. Rather its degree of presence or absence is a function of (i.e. emerges from) the interactive properties and activities of its constituent components.

Sociotechnical approaches, such as those described by Carayon et al. (2015), argue that safety emerges as a continuous function of the interactions between the numerous social and technical elements that characterise complex human-machine and organisational systems. In essence, safety can be operationally defined as the level of risk of personal injury generated by (or emerging from) the complex set of interactions between sociotechnical components of a work system. In some cases these interactions are readily perceivable and frequently lend themselves to at least generally meaningful prediction or explanation in the absence of sophisticated analysis. For instance, a significant reduction in the number of appropriately trained workers performing an inherently risky activity in an industrial setting combined with a demand to meet significantly accelerated production goals can generally be expected to result in diminished workplace safety. This is an example of a fairly linear, cause-and-effect relationship, although the study of even such apparently straightforward relationships can be problematic from a laboratory or traditional field research perspective.

The fundamental value of the concept of emergence, and complexity in general, from a sociotechnical perspective is that it affords a potentially useful context within which we strive to systemically identify, describe and study the more obscure, non-linear effects of multiple, dynamically shifting interactions among large collections of system components. The ability to conceptually grasp safety-related phenomena associated with sociotechnical systems at this level of complexity holds great promise for advancing our understanding and, presumably, our ability to enact substantive improvements in existing and future systems. However, traditional behavioural research paradigms may not be sufficiently well-equipped to address phenomena at this level of complexity. Even if they could, the sorts of field studies that one might like to conduct (e.g. assessing the impact of degraded communication and reduced staffing on petrochemical process control) are often logistically impossible and ethically untenable. Instead, we wish to propose that the development of computer-based models and simulations of sufficient fidelity may offer a useful approach to the examination of safety issues associated with sociotechnical system structure and function.

## 2. Roles of modelling and simulation in supporting sociotechnical system safety

The purpose of models is not to fit the data but to sharpen the questions. (Samual Karlin 1983)

Although the use of modelling and simulation has become ubiquitous in all areas of systems engineering, including the design of sociotechnical systems, it is useful to consider the many ways in which these techniques can contribute to system safety, and the particular advantages they offer when compared to alternative techniques or methods. Any discussion of this issue needs to acknowledge that modelling and simulation serve many purposes, both theoretical and practical. Their application can span the timeline from initial concept exploration to design to system management and operation. Furthermore, they generally involve many different types of users and stakeholders, who may have different roles, responsibilities, tasks and information needs.

We begin with some general observations from the perspective of those whose primary interest or responsibility is safety. It is hardly a secret that most unsafe outcomes are not due to random factors but instead result systematically from tradeoffs between safety and other dimensions of system design and operation that are more tangible and, thus, easier to measure, include in design specifications or to include in management or maintenance plans during system operation. All of these dimensions that frequently come into competition with safety achieve much of their competitive advantage from the fact that safety remains a concept somewhat resistant to formal definition and quantitative measurement, even after many years of research and practice.

We also now live in an age where technological pushes often dominate the pull of human needs (Postman 1993), with the result that the onus for ensuring safe systems design increasingly falls on those who would caution about the potentially risky use of ever-increasing levels of sophisticated technology and automation in the workplace or on the highways, railways or in the skies. While there is little question that we live in an age of a truly impressive and, in some cases, an even amazing pace of technological innovation, it is equally clear that much of this technology is deployed in sociotechnical systems for little reason other than the fact that it is newly available or has the flair of all that is ‘cutting edge’. As such, engineers and technologists are now, more than at any time in human history, involved in creating or shaping the human ecology, the world of technological niches in which we work and live our lives.

And yet, against this backdrop of rapid change or progress we need only turn to Plato, who in his *Phaedrus* warned that ‘ … the discoverer of an art is not the best judge of the good or harm which will accrue to those who practice it’ (Plato [n.d.] 1973, 96). Today's dominant ‘arts’, in the sense intended in this passage, are now technologies and automation in their many forms – the relevant point in Plato's observation being that those now responsible for populating the world with increasingly sophisticated tools and technologies are often not those best positioned to identify the societal consequences (‘the good or harm’) of their products to those who ‘practice’ (use) them. As a result, those whose primary interest or responsibility lies in ensuring sociotechnical system safety must be able to marshal their own technologies, such as modelling and simulation tools and techniques able to predict or control safe system design and operation, which are at a level of sophistication commensurate with the engineers, financial analysts, management scientists and others with whom they seek to sit around the table when system design, operation and management decisions are made.

If this is not the case, there is an increased chance that the readily measured, modelled, prototyped or purchased will drive out concerns for safety if the factors that contribute to or detract from safety are less tangibly represented in these negotiations, cost-benefit analyses or investment or management practices. Modelling and simulation tools that do represent these factors, together with the knowledge and ability to create and use them prudently and effectively, is perhaps the most practically useful mechanism that safety advocates have available to them during the early stages of concept exploration and design to provide value in contributing to these discussions and analyses. These front-loaded efforts taken in the name of safety could, in theory at least, begin to share a greater portion of the burden of assuring safety from the rear-loaded mechanisms of government regulation and the heavy hands of lawmaking and the threat of punitive litigation.

### 2.1. Roles of modelling and simulation during the system lifecycle

Perhaps the most salient feature of Figure 1 in the introduction of this paper, depicting a model by Leveson (2012), is the distinction between the left and right ‘columns’ associated with system development/design and system operation. We consider the different potential uses of modelling and simulation at these different phases of the system lifecycle in turn.

#### 2.1.1. Concept exploration, system development and design

We have already discussed how including any measurable safety-related factors helps in foregrounding safety as a prime concern during early formulation of system design concepts on a par with other, often competing measures of system evaluation. What might these factors include? Safety-related factors that are prime candidates for representation in modelling and simulation are the staffing and training requirements or levels imposed by particular system design concepts or operational concepts. For example, Naikar (2006) presents a sociotechnical system modelling approach that can ‘identify training needs and training-system requirements, evaluate alternative system design proposals, develop team designs, and identify training strategies for managing human error’ (423). This model, based on cognitive work analysis or CWA (Vicente 1999), is not in itself in computational form, but could presumably be given a computational realisation using the techniques provided by Hajdukiewicz and Vicente (2004) and others (e.g. Read et al. 2014). These techniques transform CWA results into a set of procedures for safe system operation and could be used as the basis of a computational model of the human operator. This model could be used as a predictive simulation for performing sensitivity analyses to assess the impact of system design factors on resulting system safety.

Another set of safety-related design issues that modelling and simulation should ideally be able to inform is how risk and the responsibility for safe levels of system operation may be influenced by different allocations of functionality to humans versus machines or automation, a form of *joint optimisation*, a concept central to sociotechnical systems approaches (e.g. Challenger et al. 2013; Dainoff 2009; Kasvi et al. 2000). These tradeoffs, and especially how risk and responsibility are collectively shared by both human and technological components of sociotechnical systems, have been nicely illustrated by Mindell (2008), in the context of NASA's Apollo space programme:

Throughout the socio-technical system that was Apollo, skill, experience and risk migrated across human and machine boundaries. The social and the technical traded off, or complemented each other, made up for each other's' weaknesses. In the real-time pressure of a lunar landing, an extensive social network of engineers focused on two men and a computer in an air-conditioned bubble, sitting on top of a rocket engine with a telescope and a control stick. (235)

While we are as yet unaware of a mature modelling and simulation technique for examining these types of issues in detail, advances in the ability to formally (mathematically, computationally) model human-automation interaction (e.g. Kirlik 1993; Degani and Heymann 2002) show promise for eventually bringing human/automation dynamics and safety-related tradeoffs within the realm of modelling and simulation to support sociotechnical system safety.

While it is easy to imagine other aspects of safety-related design features being represented in modelling and simulation for the purpose of sensitivity analysis and performance prediction, in this brief overview we instead turn our attention to an important class of quite different cognitive activities modelling can support during the design process. These activities can be usefully contrasted with the previous discussion by highlighting that the purpose is to serve as cognitive and social (teamwork) aids to a working design team, in the form of cognitive artefacts that help externalise cognition, facilitate communication and serve as a repository for previously made design decisions or commitments. Suchman (2000), for example, in a thoughtful and detailed analysis in her article ‘Embodied practices of engineering work’, provided an account of professional engineering practice that ‘emphasizes the multiplicity of media and associated objects involved in the work of engineering on the one hand, and their integration in practice into a coherent field of action on the other’. It should probably not come as a surprise that at a professional level, engineering design is characterised by many of the artefacts and trappings of any other creative design profession, one in which external media play an intimate role not solely as documentation, but as sources for ideation, communication among a design team and as a basis for a coherent coordination of the team's activities. Other types of models that are characteristically used in early stages of sociotechnical system design include computer-supported tools for storyboarding, multimedia envisioning and animation, the creation and modification of use-cases that help designers to concretise otherwise abstract design concepts and so forth.

#### 2.1.2. System operation and management

We now turn our attention to how modelling and simulation could be brought to bear to aid in ensuring the safe operation of sociotechnical systems during operational phases. One promising avenue for future development in this area is to provide bridge linking quantitative metrics associated with system operation and quantitatively-based indicators of safety culture. For instance, von Thaden and Gibbons (2008) have developed and evaluated a ‘Safety Culture Indicator Measurement System’ or SCISMS that has been widely distributed throughout the US aviation industry to quantitatively evaluate sociotechnical system safety in an attempt to prevent or reduce the frequency of unsafe acts. Though currently survey-based, there would potentially be great value in the development of quantitative metrics based on validated safety culture predictors, such as those captured in the SCISMS that could be entered into a computational simulation of sociotechnical system performance to maintain a continuous ‘dashboard’ measure of operational safety during system evolutions. Exactly how safety culture metrics would be determined from readily available measures (e.g. absenteeism, quality and production metrics) has yet to be worked out, but capitalising on the large and growing literature on safety climate and culture (e.g. Zohar 2010) and particularly predictive, quantitative metrics emerging from this research seems to be a ripe area for transitioning into sociotechnical system modelling and simulation tools.

Perhaps, obviously, modelling and simulation should also be able to play an important role during system operation in supporting ‘what-if’ sensitivity analyses to evaluate the efficacy of proposed changes to management policies. A concrete example of such a model, focused on shedding light on the particularly tenuous relation between production pressure and safety, has been provided by Cowing, Paté-Cornell, and Glynn (2004). In their own words:

A number of accidents, for example, the loss of the space shuttle Challenger and of the Piper Alpha oil platform, have occurred because upgrades and/or maintenance operations were delayed in order to meet production goals or deadlines. … [We] illustrate the use of mathematical and engineering models to support consistent choices of general operations policies and of short-term management options, based on a long-term assessment of their effects on the system's productivity and safety, and a long-term vision of the consequences of immediate decisions. (269)

Finally, we note that another almost inevitable aspect of system operation that modelling and simulation tools should support is the process of learning from failure (errors, incidents, accidents, catastrophes). These tools can assist in this domain by providing conceptual and computational frameworks enabling these events to be mined for lessons learned. The relevant modelling and simulation users here are both agencies and industries involved in actual forensic activities and sociotechnical system researchers and scholars seeking to contribute toward an improved fundamental understanding of sociotechnical system safety.

## 3. Current modelling and simulation capabilities

As discussed earlier, sociotechnical models for safety outcomes are complex and will require advanced approaches to assist in: (1) concept exploration, system development and design; (2) system operation and (3) experimental examination and analysis. One of the prominent approaches to achieve the purposes described earlier is computer simulation. A simulation is essentially a computer-based imitation of the real world to enable experimentation and analysis to derive understanding of the real world. The object of imitation is typically a system of interest to the modeller and the modeller's customers or stakeholders. In the case of empirical research, of course, the model has quite a different purpose – that being to uncover general principles of sociotechnical system structure and behaviour.

The system being modelled can be a manufacturing line, a local community, the electricity market or the global climate system. Within the context of the current discussion, the system of interest is a workplace sociotechnical system that has safety-related outcomes. Due to the multifaceted nature of a sociotechnical system, a simulation model can easily become too complex to understand. Thus, it is important for the model development process to be guided by clear purposes so that the ensuing product is as simple as possible, but detailed enough to capture all variables of interest.

There are a variety of simulation approaches in use today, and they are used both for system design and empirical analysis purposes. This section will present an overview of four main simulation approaches: (1) discrete event simulation, (2) system dynamics modelling, (3) agent-based simulation and (4) the hybrid approach. Several attempts at developing simulation models of workplace safety are then summarised, and the section then ends with a discussion of the current state of the art in modelling sociotechnical systems for safety outcomes and their limitations.

### 3.1. Simulation approaches

A simulation approach has the following fundamental components: a time-advance mechanism, a representation scheme for the simulated variables and an updating mechanism to update the values of the variables over time. A time-advance mechanism determines how the simulation advances in terms of time. There are two common forms of time-advance mechanism: the *next-event* approach and the *fixed-increment* approach. In the *next-event* approach, the computer will determine the time of all future events and advance the simulation to the most imminent next-event. At each time step, the values of the variables will be changed based on the updating mechanism, which is usually a set of equations or rules. In contrast, the *fixed-increment* approach will advance the simulation clock at fixed time intervals, but it may have a similar set of updating mechanisms. The choice of representation is dependent on the modelling paradigm of the approach. For example, one approach may be focused on modelling countable objects, while another approach may disregard individual objects and only keep track of aggregated variables. The subsequent discussion will compare and contrast the different simulation approaches based on these fundamental components.

#### 3.1.1. Discrete event simulation

**Figure 2:**
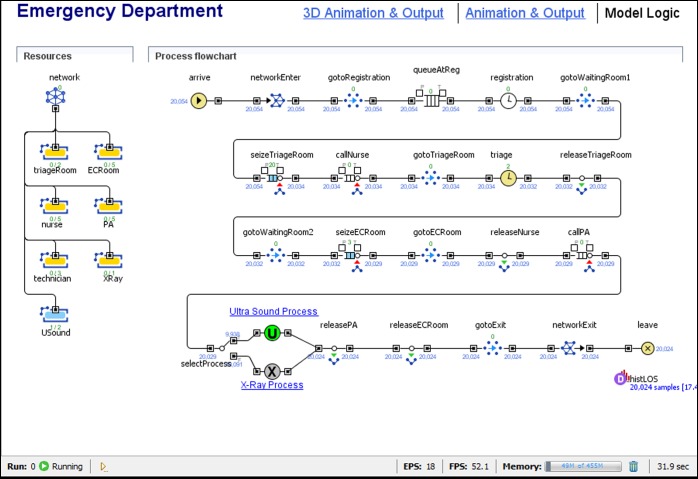
A discrete event simulation model of an emergency department (AnyLogic^TM^).

Discrete event simulation (DES) is probably one of the most commonly used simulation approaches (e.g. Banks et al. 2009). DES is commonly used to analyse processes such as a manufacturing line or a service line (see Figure 2 for an example). A DES model has a set of processes or procedures as in a flow chart. Entities are another important component of a DES model. An entity can be a person, a resource, an object of interest, a transaction or an organisation. The entities are characterised by attributes such as delay time and arrival time and the value of the attributes can be stochastic and defined by a probability distribution function. Each entity will either move through the processes or be tied to a specific step within it. Statistics such as average service time, queue length and resource utilisation are calculated as the simulation progresses. The DES approach uses the *next-event* time-advance approach, which reduces computational cost significantly as compared to the *fixed-increment* time-advance approach.

#### 3.1.2. System dynamics simulation

**Figure 3:**
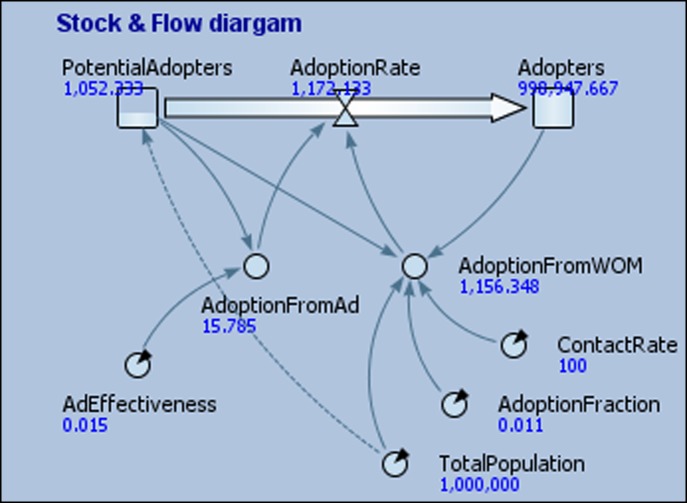
A system dynamics model for diffusion of a product (AnyLogic^TM^).

System dynamics (SD) is a simulation modelling approach that has its roots in engineering and business process modelling (e.g. Sterman 2000). At its core, an SD model is a set of differential equations. However, these models are usually represented in the diagrammatic form of stocks, flows, arrows and auxiliary variables, with the differential equations embedded within the SD model (see Figure 3 for an example). The stocks are the important variables of the model. Stocks are aggregated variables that function like pails of water with contents that are increased or decreased by the flows. The rate of change of the stock is dependent on the flow rates and the flow rates are dependent on the auxiliary variables or stocks linked to the flow rates via arrows. Each auxiliary variable will have an associated mathematical equation or logic rule (e.g. an If-Then statement). One of the key areas of emphasis of an SD simulation is the existence of feedback loops, where variables and/or stocks are connected such that a change in any of the variables or stocks in the loop can trigger a series of changes in the loop and a subsequent change in the same variable. These feedback loops can either be reinforcing (e.g. a vicious cycle) or balancing (stabilising). The combination of reinforcing and balancing loops is part of what makes the behaviour of the system complex. In contrast to DES, SD uses the *fixed-increment* time-advance approach.

#### 3.1.3. Agent-based modelling

**Figure 4:**
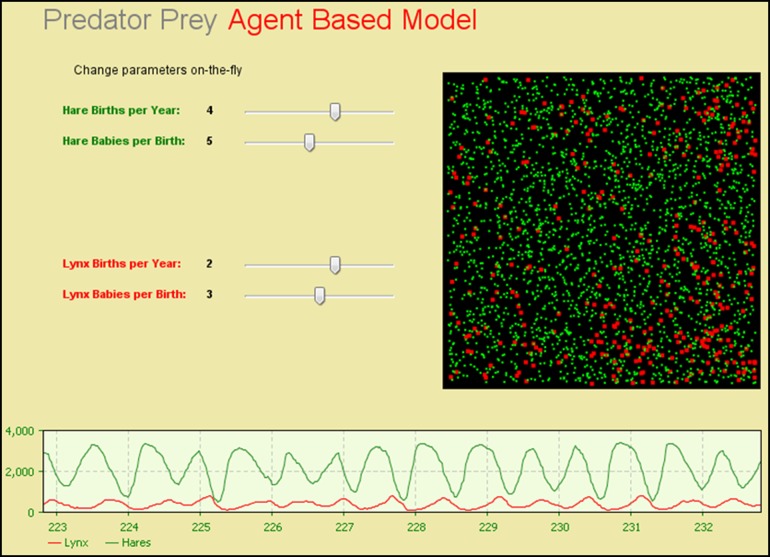
The predator-prey agent-based model (AnyLogic^TM^).

Agent-based modelling (ABM) is a comparatively new simulation approach. Similar to DES, ABM is focused on entities. Each agent is essentially an entity, but unlike DES entities, an agent has embedded rules that allow these agents to be more interactive. For example, an agent may be able to make an independent decision based on inputs from other agents and the environment. The ABM approach is more of a *bottom-up approach* in which the modeller focuses on the characteristics of the agents and not the structure of the bigger system as in the case of DES and SD models. The ABM is also suited to modelling the heterogeneity of a population, where different groups of agents can have different attributes and interactive rules. Thus, the complexity of an ABM is derived from the interactions between the agents, and the model facilitates the understanding of possible emergent behaviour of the system. The time-advance mechanism for ABM can be both *fixed-increment* and *next-event*. ABM is known for its flexibility in modelling complex social issues. Figure 4 shows the predator-prey agent-based model (Macal and North 2009).

#### 3.1.4. Hybrid simulation

**Figure 5:**
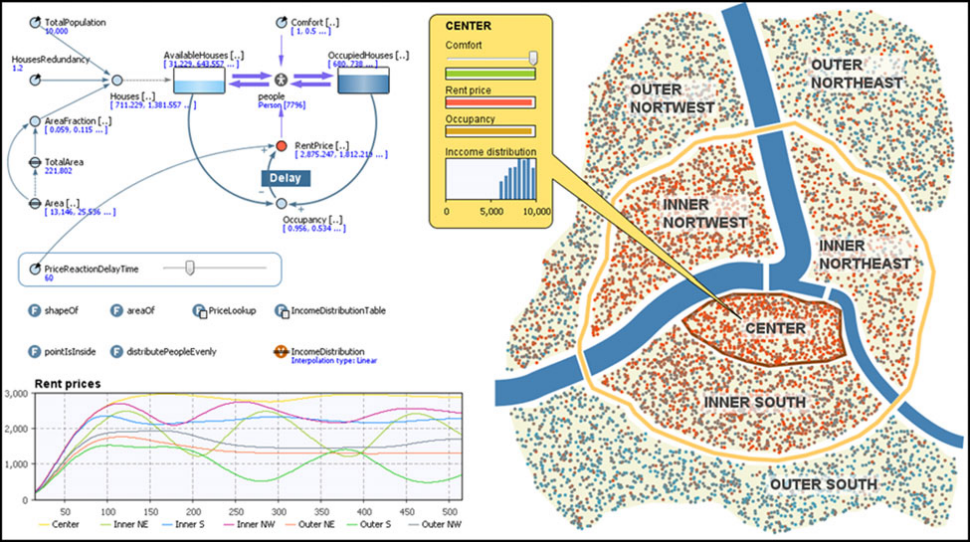
Example of an ABM-SD hybrid simulation model (AnyLogic^TM^).

In an attempt to reduce several of the shortcomings associated with each of the different methods described above, an integrated approach has been explored that has resulted in the development of so-called hybrid models. For instance, as highlighted by Lattila, Hilletofth and Lin (2010) (see Figure 5), hybrid ABM-SD models have been created to reduce the difficulties in handling heterogeneity, lack of data and to improve the flexibility of SD models. It should be noted that it is possible to use SD to model agents by using arrays for stocks and auxiliary variables. This approach is known as the agent-oriented SD approach. By using arrays, it is possible to have an agent-oriented SD model that allows each cell in the array to contain different values for different attributes of agents. However, the use of an array can easily become unwieldy when the number of agents and agent attributes are large.

### 3.2. Existing simulation models for safety

A search on the ISI Web of Knowledge database revealed seven existing simulation models for safety. Even though this search cannot be considered to be comprehensive, we believe that these seven papers provide a reasonable indication of current trends in the application of simulation techniques in safety. Each paper's research aims, the type of study and the nature of the simulation technique used are summarised in Table 1.

**Table 1:** Existing simulation models for safety.

No.	Author(s), date	Research aim	Type of study	Simulation technique
1	Rudolph and Repenning (2002)	‘… develop a general theory of how an organizational system responds to an on-going stream of non-novel interruptions to existing plans and procedures.’	Theory development	System dynamics
2	Cooke (2003)	‘This article describes a system dynamics analysis of the 1992 Westray mine disaster in Nova Scotia, Canada. It examines the causal structure of the Westray system, including relationships that could have led to conditions that caused the fatal explosion at the mine.’	Case study	System dynamics
3	Cooke and Rohleder (2006)	‘… to provide a theoretical basis for incident learning systems and provide motivation for managers to consider their implementation.’	Theory development	System dynamics
4	Sharpanskykh and Stroeve (2011)	‘An agent-based approach proposed in this paper focuses on modeling and analysis of safety culture in particular and addresses an existing gap between safety culture and organizational structures and processes.’	Methodological development	Agent-based modelling
5	Owens et al. (2011)	‘The general dynamic structure of procedure rework is described and the results of a study of procedure rework in NASA's Space Shuttle Mission Control are used to highlight implications of the structure's dynamic behavior for the investigation and utilization of these archetypes.’	Theory development	System dynamics
6	Feola et al. (2012)	‘… to investigate why PPE [personal protective equipment] underuse is so “rigid” against change in the study area. In so doing, the model is expected to uncover and represent the social processes underlying PPE misuse, and to support the identification of intervention strategies and their evaluation.’	Policy analysis or problem solving	System dynamics (agent-oriented)
7	Shin et al. (2014)	‘This paper aims to develop a system dynamics-based model of construction workers' mental processes that can help analyze the feedback mechanisms and the resultant dynamics regarding the workers' safety attitudes and safe behaviors.’	Theory development	System dynamics

In the first of these studies, Rudolph and Repenning (2002) developed a system dynamics model to evaluate the impact of a stream of non-novel interruptions on an organisation. Their model showed that a system can over-accumulate interruptions and shift from a resilient, self-regulating regime to a fragile and self-escalating regime that amplifies the impact of the interruptions, including risks to safety. However, the SD model they developed and reported on has a relatively high level of abstraction and does not identify specific agents or entities and their potential impact on safety. The model shows that as the number of interruptions accumulates the level of stress increases. When stress increases, it has both negative and positive impacts on the resolution rate of interruptions. If the negative impact dominates, the system will spiral into a disaster.

Cooke (2003) developed an SD model to describe lessons learned from the Westray mining accident of 1992 in which 26 miners lost their lives in an underground methane explosion. The SD model developed in this study, in contrast to that used by Rudolph and Repenning (2002), is very complex with several sub-systems, including production, mine capacity, human resources, and safety. Its primary utility, according to Cooke, was in its ability to provide a common stakeholder mental model of the processes and constraints that contributed to the disaster, thereby providing a useful means for identifying underlying causes.

Cooke and Rohleder (2006) modified Cooke's 2003 model to include the organisational memory of lessons learned from past accidents. Their intent was to combine aspects of Perrow's Normal Accident Theory (1984) and Rochlin's High Reliability Theory (1993) to model an organisational response system in which precursor events, or safety-related incidents, are used as the basis for instruction and planning to combat organisational complacency and promote effective learning. As with Cooke's 2003 model, Cooke and Rohleder argue that the greatest utility of their model involves its ability to synchronise stakeholders' mental models about the factors that promote accidents and the viability of potential methods for their prevention. Finally, it is important to note that both Cooke (2003) and Cooke and Rohleder (2006) factored safety-related variables such as unsafe acts, safety commitment and production pressure into their models.

In contrast to the three papers discussed so far, Sharpanskykh and Stroeve (2011) used an agent-based model (ABM) to investigate an air navigation service provider's safety culture. The focus of the study was on the reporting and investigation of safety-related incidents and the monitoring of safety performance and improvement. In the ABM, each agent was modelled to have the ability to hold beliefs about the environment that they work in and about other agents in the environment. Based on those beliefs, the agent decides whether to report a safety occurrence or not. Aggregated indicators of safety culture were then obtained from the simulation runs. In addition, sensitivity analyses were conducted. The results of the simulation model were validated by comparing with an actual safety culture survey and workshop.

Owens, Leveson and Hoffman (2011) integrated a complex system dynamics production model, designed and successfully applied to assess the economic impact of rework orders on US Navy ship production (Cooper 1980) with the system dynamics model developed by Rudolph and Repenning (2002) described above. Their intent was to understand how these two models could be integrated to assess the impact of rework on the likelihood of disaster occurrence. As both initial models used system dynamics techniques, Owens et al. also used a system dynamics methodology. Through examination of data derived from Space Shuttle missions, the authors were able to demonstrate a clear linkage between the system dynamics of rework procedures and those that impacted ‘disaster dynamics’.

Feola, Gallati and Binder (2012) used an agent-oriented SD model to study the issue of misuse of personal protective equipment (PPE) among pesticide applicators in Colombia. A model was developed based on data collected from a farming region in Colombia. The model was then used to investigate how different interventions could be applied to reduce the problem of PPE misuse. The authors indicated that an agent-based modelling approach was not necessary because there was minimal interaction between farmers (agents). The agent-oriented SD approach was determined to be sufficient to model the heterogeneity of the farmers.

Finally, Shin et al. (2014) have recently applied system dynamics modelling to assess mental process factors underlying unsafe acts by construction workers. ‘Unsafe acts’ are a behavioural aspect of safety widely considered to have a significant impact on the frequency and severity of accidents in many industries, an idea stemming from the earliest days of safety science (e.g. Heinrich 1959). The authors' results suggest that SD models can be usefully applied as both a descriptive tool (i.e. to promote development of common stakeholder mental models, as discussed above) and to evaluate the potential effectiveness of candidate interventions such as, in this case, incentives for safe behaviours and improvements in safety-related communications.

Out of the seven articles, only one paper used an agent-based modelling approach. All other models used a system dynamics methodology and none of the models utilised discrete event simulation. In contrast to the SD and ABM approaches, DES does not readily facilitate the investigation of so-called ‘softer’ factors such as cognition of safety-related information and individual decision.

Based on the papers identified in Table 1, it appears that an ABM or at a minimum, an agent-oriented SD model may provide greater potential in discovering emergent properties of the system. ABM also offers greater flexibility in accounting for social interactions between agents and the heterogeneity of agents attributes. However, from a sociotechnical systems perspective, ABM might not be the optimal approach. It should be noted that DES and SD models have a longer tradition of modelling technical or procedural components. Thus, it is suggested that sociotechnical systems should adopt a hybrid simulation approach to reflect both the social and technical aspects. The social aspect should perhaps be modelled using ABM and the technical aspect modelled using DES and/or SD. However, the internal cognitive processes of each agent can still be modelled using an embedded SD model.

## 4. Conclusion: priorities for sociotechnical system modelling and simulation

As other papers in this special issue have highlighted (e.g. Carayon et al. 2015; Flach et al. 2015) our current conceptual understanding of complex sociotechnical systems is superior to our capability to develop corresponding computer-based models and simulations The potential utility of such tools has been described above, but to summarise in terms of what an effective computer-based model or simulation should afford the scientific, engineering and management communities, we propose the following. First, effective models and simulations should help enable effective system design, deployment and sustainment decisions by supporting accurate, shared mental models of system structure and dynamics, taking into account critical social-organisational and technical system components and their interactions. Second, the scientific study of sociotechnical systems, whether with respect to workplace safety (this paper's principal area of concern) or any other emergent system attribute, requires models and simulations that accurately capture key elements of system complexity, including emergence, adaptation and resilience (or, its opposite, brittleness).

Our understanding of these key parameters of sociotechnical systems is likely to grow substantially as modelling efforts begin to explore the space identified by conceptual models and corresponding empirical efforts. This will promote, one hopes, a durable feedback loop in which converging approaches elaborate upon and extend the findings of others. As argued by Waterson et al. (2015), it is unlikely that any single design approach or empirical method will be able to independently account for the full scope of factors impacting the behaviour of sociotechnical systems. Modelling and simulation will remain one of many complementary approaches, albeit one with potentially significant advantages in terms of ease of examining system design and deployment tradeoffs, for instance.

There is also a case to be made for leveraging models of adaptive complexity from other social science areas, as well as those from physics and the biological sciences. While there is little *a priori* reason to assume that all complex, adaptive systems (i.e. social, technical, biological, etc.) behave according to common, specific principles, there are those who make compelling arguments in that vein (e.g. Berkes and Folke 2000; Holland 2012). There may be great value in examining the potential similarities and differences in computational approaches to complexity in these disparate domains. Evolutionary psychologists, for instance, might have very useful perspectives on the adaptive evolution of sociotechnical systems based on their study of the evolution of complex biological and social systems.

Contemporary sociotechnical systems present many challenges to our abilities to envision, study and, eventually, comprehend them. The intricacy of their structural and dynamic properties poses significant problems for system designers who often face high levels of uncertainty when attempting to understand the potential impact of changes to their design. Modifications to individual system elements or sub-systems within highly interconnected sociotechnical systems will impact other elements and sub-systems, often in ways that cannot be accurately predicted. Overlooked or unintended consequences of changes to such systems are a frequent cause of safety risks and overall system malfunction.

Computer-based models and simulations are unlikely to ever be able to convey information about all possible potential consequences of a design modification. Fundamental limitations arise at the level of not being able to completely and accurately predict the nature and impact of all factors endogenous to the system which may nevertheless exert powerful influences on its behaviour. However, models and simulations that provide the ability to conduct multiple, iterative test runs of system performance in operationally relevant scenarios should be able to provide designers with useful information about the probabilities of various adverse consequences, possibly even illuminating hitherto unforeseen, unintended consequences. Ultimately, any system developer will have to converge on a less-than-perfect design, simply due to the fact that no design can be free of adverse tradeoffs or unforeseen negative consequences. However, models that can support the assessment of design tradeoffs by providing reliable information about ‘downstream’ system performance consequences of design decisions will be very helpful.

Researchers wishing to study sociotechnical systems are confronted with a somewhat different problem. While the system designer wishes to accurately envision and understand how an *individual* system can be expected to behave, the researcher seeks to understand how sociotechnical systems *in general* behave, and how changes to their structural and dynamic properties influence their current and future behaviours. At present, this is a daunting task. Laboratory-based experimentation, while offering a high level of experimental rigor and control, generally cannot fully replicate the complex chain of interdependent processes that influence the behaviour of sociotechnical systems. Field research, while capturing behaviour in its natural context, generally does not afford the ability to modify key system attributes in order to gauge the impact on key aspects of performance. In essence, we currently do not have the means to study questions that involve examining the impact of structural and dynamic changes within sociotechnical systems on safety and other performance-relevant metrics.

The conduct of ecologically valid research in this field is reliant on the development of empirical tools that do not compromise the fidelity of the phenomenon under investigation in order to conform to the demands of traditional research paradigms. Modelling and simulation affords a particularly effective means for aiding both the system designer and the safety researcher in their need to better understand complex sociotechnical systems. In many respects, we are not far removed from the realisation of that capability.

## Footnotes

^4.^ Note that in the countries of the European Union, the term ‘human factors integration’ (e.g. Tainsh 2004) is more commonly used than ‘human-systems integration’, although the principles underlying both are largely identical.

^5.^ In essence, the whole of the planet's social ecosystem comprises a vast sociotechnical system. When viewed from this perspective the ripple effects of any particular sub-system's activities can have very diffuse and often unpredictable influences on other sub-systems as well as on the system as a whole, a notion that is a mainstay of complexity theory (c.f., Miller and Page 2007).
